# A Treatise on Materia Medica

**Published:** 1882-04

**Authors:** 


### BOOK REVIEWS, NOTICES, Etc.

A TEXT-BOOK OF MATERIA MEDICA: Characteristic, Analytical and Comparative. 2d edition. By *A. C. Cowperthwaite, M. D., Ph. D.*, etc., etc. Pp. 576. Chicago: Duncan Brothers. 1882.

In less than six hundred pages Prof. Cowperthwaite gives a clear, but necessarily brief, synopsis of the characteristic symptoms of nearly three hundred drugs. Some remedies, not in the first edition, have been added; others, thoroughly revised, and many new "comparisons" added. These comparisons are a new and special feature of Prof. Cowperthwaite's book. We think the space occupied in giving the "therapeutic range" of each drug could have been put to better use. For in spite of the author's warning, given in his preface, this part is apt to be abused. The printer's work has not been done as well as could be desired; the paper is too thin and type too small.

